# Non-calcifying Langerhans Cell Rich Variant of Calcifying Epithelial Odontogenic Tumor and Amyloid Rich Variant of Central Odontogenic Fibroma: A Unique Entity or a Spectrum?

**DOI:** 10.3389/froh.2021.767201

**Published:** 2021-10-25

**Authors:** Chih-Huang Tseng, Pei-Hsuan Lu, Yi-Ping Wang, Chun-Pin Chiang, Yi-Shing Lisa Cheng, Julia Yu Fong Chang

**Affiliations:** ^1^Division of Oral Pathology and Maxillofacial Radiology, Kaohsiung Medical University Hospital, Kaohsiung, Taiwan; ^2^Oral and Maxillofacial Imaging Center, College of Dental Medicine, Kaohsiung Medical University, Kaohsiung, Taiwan; ^3^Graduate Institute of Clinical Dentistry, School of Dentistry, National Taiwan University, Taipei, Taiwan; ^4^Department of Dentistry, National Taiwan University Hospital, College of Medicine, National Taiwan University, Taipei, Taiwan; ^5^Graduate Institute of Oral Biology, School of Dentistry, National Taiwan University, Taipei, Taiwan; ^6^Department of Dentistry, Hualien Tzu Chi Hospital, Buddhist Tzu Chi Medical Foundation, Hualien, Taiwan; ^7^Department of Diagnostic Sciences, Texas A&M University College of Dentistry, Dallas, TX, United States

**Keywords:** odontogenic tumor, calcifying epithelial odontogenic tumor, central odontogenic fibroma, non-calcifying Langerhans cell rich variant CEOT, amyloid rich variant central odontogenic fibroma

## Abstract

Overlapping clinicopathological features of non-calcifying Langerhans cell rich variant of calcifying epithelial odontogenic tumor (NCLC-CEOT) and the amyloid rich variant of the central odontogenic fibroma (AR-COF) have been recognized recently. It is still under debate whether these two diseases are indeed one unique disease entity or belong to CEOT and COF, respectively. To clarify this issue, we have performed a literature review to compare the similarities and differences in clinicopathological features among NCLC-CEOT, AR-COF, classic CEOT, and classic COF. We aimed to investigate whether NCLC-CEOT and AR-COF might be the same and one distinctive disease entity, or a variant (or variants) of either CEOT or COF; or whether COF, NCLC-CEOT/AR-COF, and CEOT represented a histopathological spectrum of one disease. Our results indicate that NCLC-CEOT and AR-COF cases share many similar clinicopathological features. Thus, we suggest that they are the same disease entity. Due to nearly no reported recurrence of NCLC-CEOT/AR-COF cases, the conservative surgical treatment is appropriate. The NCLC-CEOT/AR-COF cases show some overlapping clinicopathological features with COF rather than the CEOT cases. However, differences in the clinicopathological features are still recognized among the NCLC-CEOT/AR-COF, COF, and CEOT cases. Future research, particularly molecular biological studies, may further elucidate their relationships and assist proper classification of the NCLC-CEOT/AR-COF cases.

## Introduction

**Figure 1:**
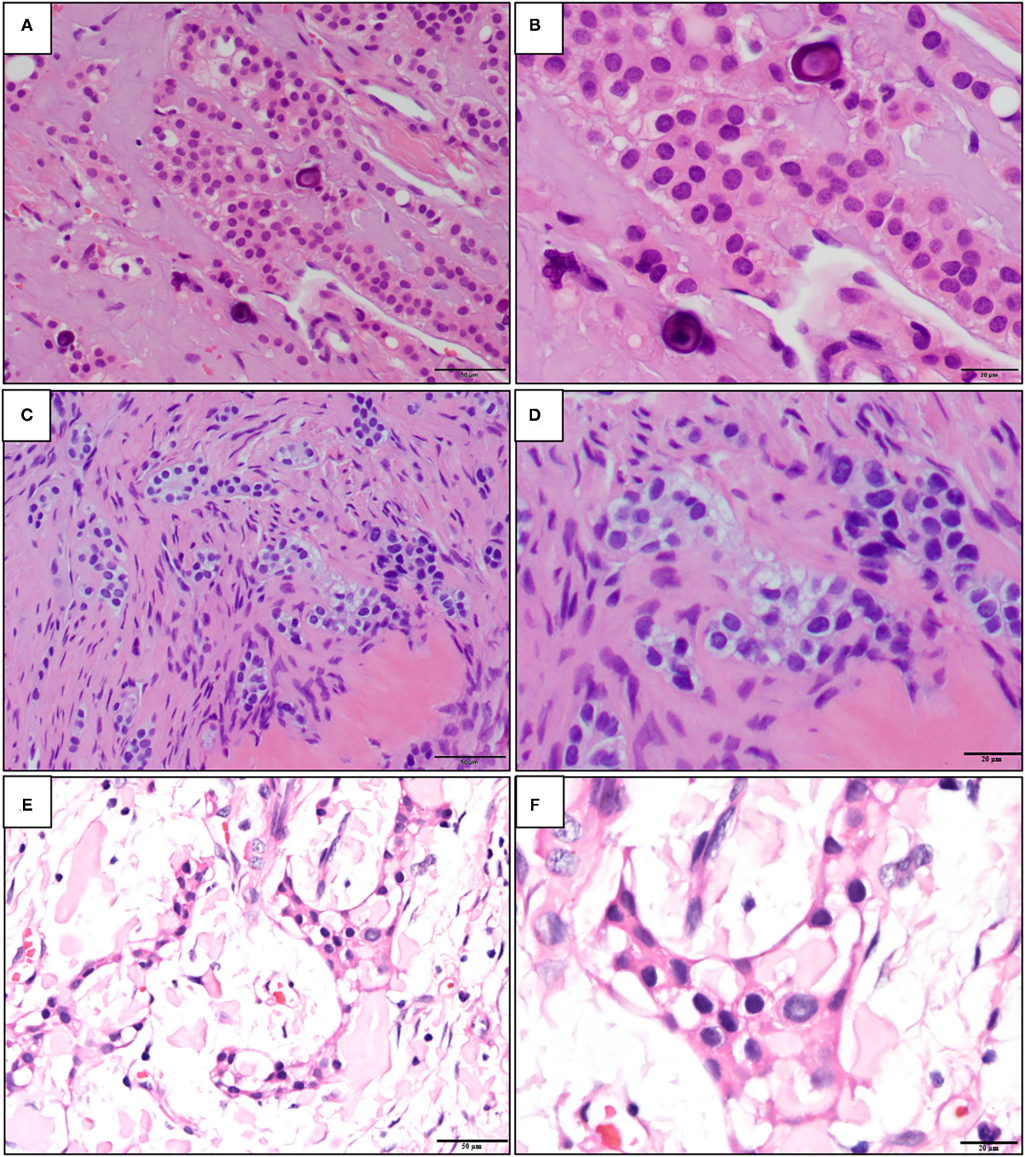
The representative histopathological photographs of **(A,B)** classic calcifying epithelial odontogenic tumor (CEOT); **(C,D)** central odontogenic fibroma (COF); and **(E,F)** non-calcifying, Langerhans cell rich CEOT (NCLC-CEOT) [Hematoxylin and eosin (H&E); **A,C,E**: 200×; **B,D,F**: 400×].

A calcifying epithelial odontogenic tumor (CEOT) has been first recognized as a distinct entity by Pindborg in 1955 [1, 2], although few cases had been described using different diagnoses earlier [3, 4]. Thus, CEOT has also been known as a “Pindborg tumor.” CEOT is mainly an intraosseous neoplasm, but some extraosseous cases have been reported. The characteristic histomorphological features of CEOT are the sheets of polyhedral epithelial cells showing distinct cell borders and intercellular bridges admixed with a homogeneous substance, which is interpreted as amyloid or amyloid-like products, and calcified materials in the form of concentric Liesegang's rings [3, 5] (Figures 1A,B). The CEOT with epithelial cells showing various degrees of cellular and nuclear hyperchromatism and pleomorphism, prominent nucleoli, and some mitotic figures are sometimes misdiagnosed as intraosseous carcinoma [3]. These histomorphologic changes have been described in the previous studies [2, 3, 6, 7]. The clear cell variant has first been mentioned by Abrams and Howell [8] in 1967. The cellular abnormalities and glandular-like configuration have also been reported early in the 1960s [3, 7]. Langerhans cell variant of CEOT has started to be reported from the 1990s [9].

The early description of the odontogenic fibroma (OF) was started by Thomas and Goldman in 1946 [10]. However, the diagnostic criteria for OF were established in the first WHO classification of odontogenic tumors in 1971 [11]. OF is classified as a mesenchymal odontogenic tumor that is composed of mature fibrous connective tissue with variable amounts of odontogenic epithelium with or without deposition of calcified materials (Figures 1C,D). Both central (intraosseous) and peripheral (extraosseous) OFs have been reported. The odontogenic epithelial islands or strands dispersed in the fibrous connective tissue stroma of OF are usually inactive-looking. The hard tissues with features of dentinoid or cementum-like calcifications associated with odontogenic epithelium can also be found. The WHO recognizes two subtypes of central OF (COF), namely, epithelial-rich or WHO type and epithelial-poor or simple type. Other rare variants, such as ossifying COF, COF associated with giant cell lesions, granular cells, and amyloid have been documented [12–15].

Both the CEOT and COF are rare odontogenic tumors. Based on the major components of the tumors, the CEOT is currently classified as an epithelial odontogenic tumor and instead, the COF is classified as a mesenchymal odontogenic tumor. In the 2017 WHO's classification [16], the CEOT is defined as a benign epithelial odontogenic tumor that secretes an amyloid protein that tends to calcify. The definition for COF is a neoplasm of mature fibrous connective tissue, with variable amounts of inactive-looking odontogenic epithelium with or without evidence of calcification. Therefore, the classic CEOT and COF cases have their distinctive characteristic histopathologic features and accurate diagnosis should not be a problem. However, some variants of CEOT and COF had been reported in the literature during the past decades. Among the variants, the overlapping clinicopathological features of non-calcifying Langerhans cell rich variant of CEOT (NCLC-CEOT) [9, 16–20] (Figures 1E,F) and the amyloid rich variant of COF (AR-COF) [13–15, 21, 22] have been recognized recently. Thus, the disease diagnosed as either NCLC-CEOT or AR-COF started to be considered as the same disease [13, 15, 23]. Because a predilection for the anterior to premolar region of the maxilla, disassociation with an impacted tooth, frequent palatal depression, and fewer recurrence cases are all features less commonly seen in classic CEOT, it has been proposed that this disease is better categorized as AR-COF rather than NCLC-CEOT [13, 15, 23]. However, differentiation between CEOT and COF is not always straightforward, because the non-calcifying CEOT cases have also been reported [3, 24] and thin strands or small odontogenic epithelial nests are sometimes present in CEOT either as a major or minor component [5, 6, 24]. In this study, we aimed to review the available data published in the literature and investigated whether NCLC-CEOT and AR-COF might be the same and one distinctive disease entity, or a variant (or variants) of either CEOT or COF; or whether COF, NCLC-CEOT/AR-COF, and CEOT represented a histopathological spectrum of one disease.

## Materials and Methods

This study followed the preferred reporting items for systematic reviews and meta-analyses (PRISMA) Statement guidelines [25]. A review protocol does not exist.

### Literature Search Strategies

A literature review was performed using PubMed with the search terms “calcifying epithelial odontogenic tumor,” “central odontogenic fibroma,” “Non-calcifying Langerhans cell rich calcifying epithelial odontogenic tumor,” and “central odontogenic fibroma, amyloid variant.” A literature search period was set between 1958 and June 1, 2021. Only publications in the English language were included in this analysis.

### Study Selection

**Figure 2:**
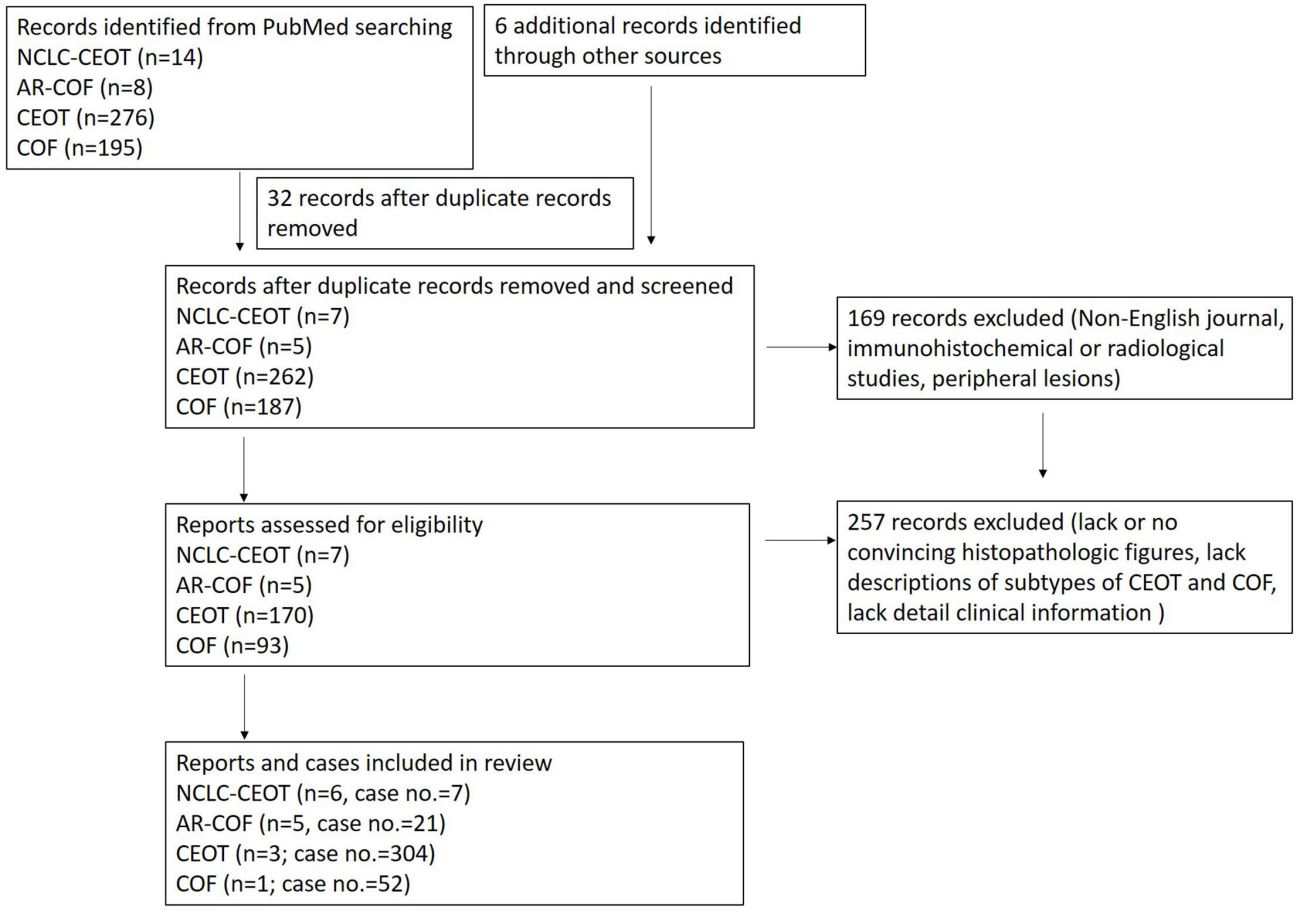
Study screening process.

The study selection process is summarized in Figure 2. In this study, the focused diseases were intraosseous NCLC-CEOT and AR-COF. All the cases from case reports or case series named NCLC-CEOT [9, 16–20] or AR-COF [13–15, 21, 22] were reviewed. The cases included in review articles focusing on NCLC-CEOT or AR-COF and review articles including information about the NCLC-CEOT or AR-COF variants were also reviewed [22–24, 26–29]. The inclusion criteria were the case reports and case series with convincing histopathological photographs for confirming the diagnosis (reviewed by two board-certified oral pathologists) and containing detailed clinical information for further comparisons. The exclusion criteria were the cases without convincing histopathological photographs; peripheral (extraosseous) CEOT or COF; and those that lack detailed clinical information for further comparison [24, 26–30]. The duplicated cases were also excluded. Two cases initially reported as NCLC-CEOT but later were included in an AR-COF case series were still listed in the NCLC-CEOT group [15, 18] in this study. A total of seven NCLC-CEOT cases from the six reports and 21 AR-COF cases from the five reports were included in Table 1. Cases 3 and 6 were previously published from our institution and these two cases were reviewed extensively based on the available information.

**Table 1:** Clinical findings in 7 non-calcifying, Langerhans cell rich calcifying epithelial odontogenic tumor (NCLC-CEOT) as well as 21 amyloid rich variant of central odontogenic fibroma (AR-COF).

**Case**	**Author/ country**	**Sex**	**Age**	**Location**	**Diagnosis**	**Clinical and radiographic findings**						**Treatment and outcome**	
						**Clinical/Palataldepression**	**Cortical bone disruption**	**Cortical boneexpansion**	**Root resorption**	**Tooth displacement**	**Impacted tooth**	**Treatment**	**Recurrence**
1	Asano [9], Japan	F	44	Maxilla, central incisor to first molar	NCLC-CEOT	Gingival swelling/NA	NA	+	+	-	-	Partial resection	NA
2	Takata [16], Japan	M	58	Maxilla, canine to second premolar	NCLC-CEOT	Loosening tooth/NA	+ (buccal and palatal)	-	+	+	-	Enucleation	No recur for 10years
3	Wang [17], Taiwan	F	52	Maxilla, lateral incisor to canine	NCLC-CEOT	Depression on palate	+ (palatal)	-	-	-	-	Partial resection	No recur for 15 years
4	Chen [18], China	F	40	Maxilla, central incisor to first premolar	NCLC-CEOT	Loosening tooth/palatal depression	+ (buccal and palatal)	-	+	-	-	Curettage	No recur for 5 years
5	Chen [18], China	M	58	Maxilla, lateral incisor to second molar	NCLC-CEOT	Swelling, loosening tooth/NA	+	-	+	-	-	Partial resection	No recur for 10 years
6	Tseng [19], Taiwan	M	24	Maxilla, canine to second premolar	NCLC-CEOT	Loosening tooth, biting pain	+ (palatal)^*^	-	+	-	-	Curettage	No recur for 6 years
7	Santosh [20], USA	F	43	Maxilla, lateral incisor to second premolar	NCLC-CEOT	Asymptomatic	-	-	+	-	-	Excision	No recur for 1.5 years
													
8	Eversole [13], USA	F	77	Maxilla, premolar region	AR-COF	NA	NA	NA	NA	NA	NA	NA	No recur
9	Eversole [13], USA	F	39	Maxilla, premolar region	AR-COF	NA	NA	NA	NA	NA	NA	NA	No recur
10	Eversole [13], USA	F	38	Maxilla, premolar region	AR-COF	NA	NA	NA	NA	NA	NA	NA	No recur
11	Eversole [13], USA	M	27	Maxilla, molar region	AR-COF	NA	NA	NA	NA	NA	NA	NA	No recur
12	Zhou [15], China	M	33	Maxilla, lateral incisor to first molar	AR-COF	loosening teeth/palatal depression	NA	NA	+	-	-	Excision	No recur for 6 months
13	Zhou [15], China	F	59	Maxilla, canine to second premolar	AR-COF	loosening teeth/palatal depression	NA	NA	+	-	-	Excision	No recur for 12 months
14	Zhou [15], China	M	38	Maxilla, canine to first molar	AR-COF	loosening teeth/palatal depression	NA	NA	+	+	-	Excision	No recur for 21 months
15	Zhou [15], China	F	32	Maxilla, lateral incisor to second premolar	AR-COF	loosening teeth/palatal depression	NA	NA	+	-	-	Excision	No recur for 12 months
16	Correa Roza [14], Brazil	F	16	Mandible, posterior	AR-COF	NA	-	+	-	+	-	NA	NA
17	Correa Roza [14], Brazil	M	55	Maxilla, anterior (canine)	AR-COF	Palatal swelling	NA	NA	-	-	-	Excision	No recur for 8 years
18	Correa Roza [14], Brazil	F	52	Maxilla, anterior (incisor)	AR-COF	Palatal depression	+	-	+	-	-	Excision	No recur for 1 year
19	Correa Roza [14], Brazil	M	35	Maxilla, anterior (incisor and premolar)	AR-COF	Palatal depression	-	+	+	-	-	Excision	NA
20	Correa Roza [14], USA	F	34	Maxilla, anterior	AR-COF	NA	NA	NA	NA	NA	NA	NA	NA
21	Correa Roza [14], USA	F	57	Maxilla, premolar	AR-COF	NA	NA	NA	+	-	NA	NA	NA
22	Correa Roza [14], Mexico	F	36	Maxilla, canine and premolar	AR-COF	Buccal swelling/Palatal depression	+	+	+	-	-	Excision	No recur, 6 months
23	Correa Roza [14], South Africa	F	60	Maxilla, incisor and canine	AR-COF	NA	NA	NA	NA	NA	-	NA	NA
24	Correa Roza [14], Chile	F	23	Maxilla, incisor	AR-COF	NA	NA	NA	NA	+	-	NA	No recur
25	Correa Roza [14], Chile	M	35	Maxilla, incisor and canine	AR-COF	Erythematous mucosa	-	+	+	+	-	Partial resection	No recur, 3 years
26	Kakuguchi [21], Japan	M	35	Mandible, canine to first molar	AR-COF	Asymptomatic/ lingual depression	+	-	+	-	-	Enucleation and curettage	No recur, 5 months
27	Ruddocks [22], USA	F	34	Maxilla (palate)	AR-COF	NA	NA	NA	NA	NA	-	NA	Recurrent by history
28	Ruddocks [22], USA	F	47	Maxilla, lateral incisor to canine	AR-COF	NA	NA	NA	NA	NA	-	NA	NA

*NA, not available*.

* *Our original paper did not mention palatal depression. After reviewing the case, palatal depression was noted*.

The classic CEOT and classic COF were defined as CEOT excluding NCLC-CEOT cases and COF excluding AR-COF cases. Since the majority of the previous reviews or case series did not separate NCLC-CEOT and AR-COF from CEOT and COF, respectively, the clinical data of classic CEOT and classic COF were mainly used in the most recent case series [14, 24, 31] or review articles [32], which particularly recognized and separated NCLC-CEOT and AR-COF variant cases from the classic CEOT and COF cases, respectively.

### Data Analyses

The clinical data, including sex, age, tumor location, teeth involved, presence of palatal depression, swelling, cortical bone disruption, cortical bone expansion, root resorption, tooth displacement, and recurrence for NCLC-CEOT, AR-COF, classic CEOT, and classic COF, were retrieved and compared. The cases labeled with “not available (NA) information” were excluded from our analysis. The differences in the clinical data among the NCLC-CEOT, AR-COF, classic CEOT, and classic COF cases were evaluated with a chi-square test. All the analyses were performed using IBM SPSS Statistics for Windows, version 20.0 (IBM Corp., Armonk, NY, USA). A *p*-value < 0.05 was considered to be significant.

## Results

### The NCLC-CEOT and AR-COF Cases Share Similar Clinicopathological Features and Most Likely to Be the Same Disease

#### Clinical Findings

**Figure 3:**
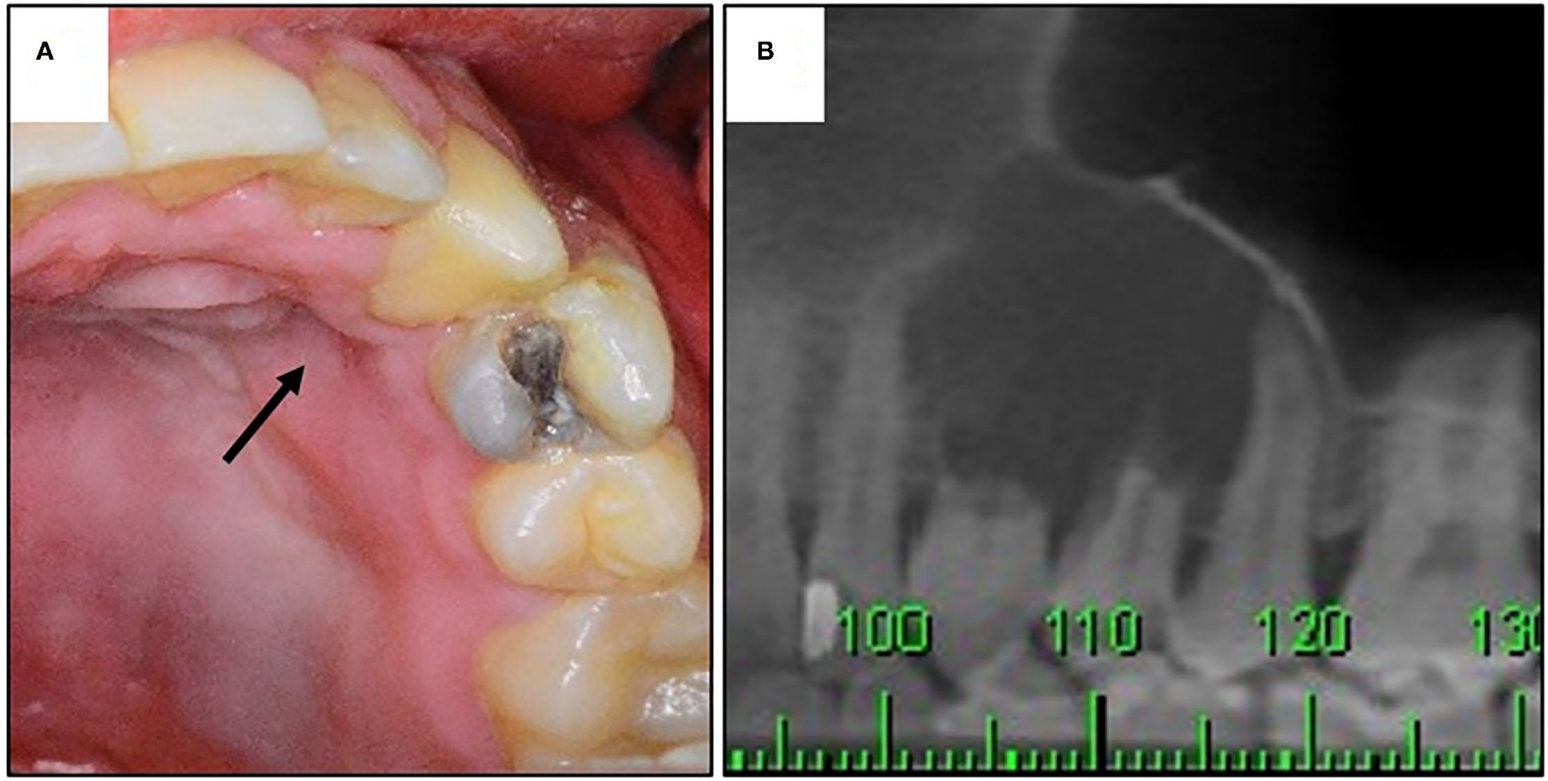
The clinical and radiographic findings of a patient with NCLC-CEOT. **(A)** Clinical picture showing palatal depression (arrow); **(B)** prominent root resorption in cone-beam CT (CBCT) image.

The clinical and radiographic features of seven NCLC-CEOT cases from six reports and 21 AR-COF cases from five reports are summarized in Table 2. The clinical picture and the panoramic view of cone-beam CT (CBCT) image of our previous reported NCLC-CEOT case (case 6 in Table 1) were shown (Figure 3) to demonstrate the common findings of the NCLC-CEOT and AR-COF cases.

**Table 2:** Comparisons of clinical findings in non-calcifying Langerhans cell rich variant of calcifying epithelial odontogenic tumor (NCLC-CEOT), amyloid-rich variant of central odontogenic fibroma (AR-COF), CEOT, and COF cases.

**Case**	**Sex**	**Age**	**Location**	**Tooth^a^**	**Clinical and radiographic findings**						**Outcome**
					**Palatal** **depression**	**Swelling**	**Cortical bone disruption**	**Cortical boneexpansion**	**Root resorption**	**Tooth displacement**	**Recurrence**
NCLC-CEOT (1, 3, 4, 5, 6, 7)	M: 3 F: 4	45.8 ± 11.1 (24–58)	Mx: Md = 7:0	A: P: M = 7:4:0	3/742.9%	2/728.6%	5/771.4%	1/714.3%	6/785.7%	1/714.3%	No recur
AR-COF (8, 9, 10, 11, 12)	M: 7 F: 14	41.1 ± 14.0 (16–77)	Mx: Md = 19:2	A: P: M = 12:12:2	8/10**^b^**80%	2/1020%	3/10 30%	3/1030%	10/14 71.4%	4/1428.6%	1/21**^c^** 4.8%
NCLC-CEOT/AR-COF	M: 10 F: 18	42.2 ± 13.5 (16–77)	Mx: Md = 26:2	A: P: M = 19:16:2	10/17 58.8%	4/1723.5%	8/17 47.1%	4/1723.5%	16/21 76.2%	5/2123.8%	1/28 3.6%
CEOT (15, 23, 24)	M: 152 F: 152	37.4 ± 18.3 (4–78)	Mx: Md = 121:182**	A: P: M = 60:76:127**	NA	13/2552%	78/199 39.2%	8/2532%	24/198** 12.1%	143/240*59.6%	20/173 11.6%
COF (10)	M: 16 F: 36	32.7 ± 15.4* (8–63)	Mx: Md = 23:29**	A: P: M = 22:10:29**	7/20**^b^**35%	9/2045%	17/48 35.4%	23/4847.9%	4/48** 8.3%	20/4841.7%	1/15 6.7%

a *Tooth: A, anterior; P, premolars; M, molars; involved areas were counted (ex. both anterior teeth and premolar teeth were involved, then both A and P would be counted.)*.

b *One lingual depression included in AR-COF group and one alveolar bone depression included in COF group*.

c Recurrence by history. *NCLC-CEOT/AR-COF vs CEOT or COF, p < 0.01; **NCLC-CEOT/AR-COF vs. CEOT or COF, p < 0.001.

The NCLC-CEOT cases occurred nearly equally in male and female patients, similar to the classic CEOT cases, and the AR-COF cases occurred predominantly in female patients with a female to male ratio of 2:1, similar to the classic COF cases. However, the gender distribution data might not be an accurate reflection due to too few cases in the NCLC-CEOT (*n* = 7) group. There was no significant difference in the mean age or age range between the NCLC-CEOT and AR-COF groups. The majority of NCLC-CEOT and AR-COF cases occurred in the maxilla with the maxilla to mandible ratios of 7:0 and 19:2, respectively. The anterior and premolar region of the maxilla were frequently involved in both the NCLC-CEOT and AR-COF cases. There was no significant difference in the tumor location or tooth involvement between the NCLC-CEOT and AR-COF cases.

Interestingly, palatal depression (Figure 3A) was frequently present in both the NCLC-CEOT (3/7) and AR-COF (8/10) cases. Cortical bone disruption rather than cortical bone expansion was more frequently seen in the NCLC-CEOT cases. However, in the AR-COF cases, cortical bone disruption and cortical bone expansion were equally present. In both the NCLC-CEOT and AR-COF groups, root resorption (Figure 3B) was frequently identified in 6/7 and 10/14 cases, respectively. A tooth displacement was occasionally seen in both the NCLC-CEOT (1/7) and AR-COF (4/17) groups. No significant differences in the clinical and radiographic findings were identified between the NCLC-CEOT and AR-COF cases. In clinical outcome, all cases either treated with conservative curettage or enucleation or radical resection showed no recurrence. One exception in the AR-COF group was a case described with a recurrent history at the time of biopsy [21]. However, no information regarding the previous lesion was provided, and no recurrence in this specific case was noted after the treatment. Therefore, both the NCLC-CEOT and AR-COF groups tended to have a good clinical outcome and nearly no recurrence was noted even after the conservative surgical treatment.

#### Histopathological Findings

**Figure 4:**
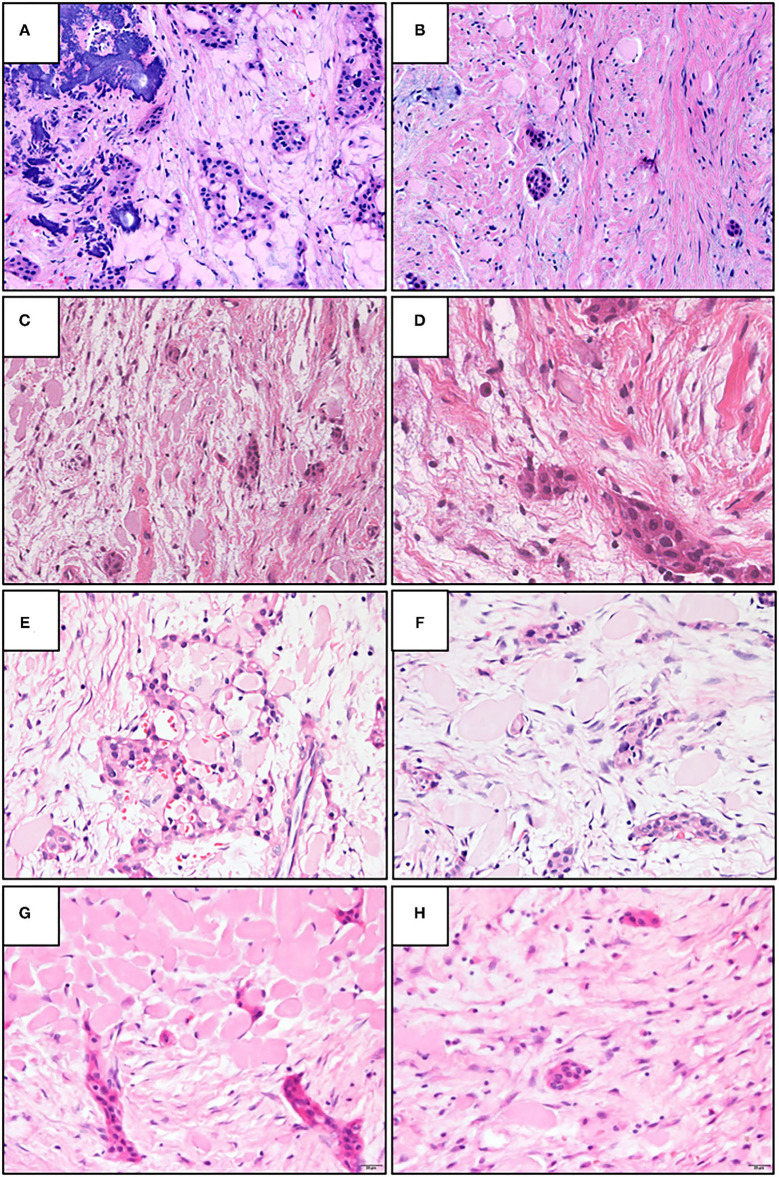
The representative histopathologic photographs of CEOT, amyloid rich variant of the central odontogenic fibroma (AR-COF), and NCLC-CEOT. **(A,B)** A classic CEOT case with **(A)** showing the classic pattern and **(B)** showing the features like an AR-COF in the periphery of the same case. **(C,D)** An AR-COF case with **(C)** showing some amyloid-like materials dispersed in classic COF stromal background and **(D)** showing intercellular bridges and mild nuclear hyperchromatism in the odontogenic epithelial islands. **(E,F)** An NCLC-CEOT case 3 exhibiting strands or nests of odontogenic epithelium and amyloid materials in the fibrous connective tissue stroma. **(G,H)** An NCLC-CEOT case 6 demonstrating a network or nests of odontogenic epithelium and amyloid materials in the loose connective tissue stroma. H&E; **A–C**: 100×; **D**: 400×; **E–H**: 200×.

Both NCLC-CEOT and AR-COF share similar histopathological features from the literature review. The tumor is composed of various numbers and sizes of epithelial strands or nests and different amounts of amyloid-like products dispersed in the mixed loose and dense fibrous connective tissue stroma. Although small odontogenic epithelial nests, which are commonly seen in the classic COF cases, can be seen in the NCLC-CEOT/AR-COF cases (Figures 4C,F,H), slightly increased strands or nests of proliferating odontogenic epithelial cells are not uncommonly seen in the NCLC-CEOT cases (Figures 1E,F, 4E). In high-power views, some of the proliferative odontogenic epithelial strands or nests show intercellular bridges. Slightly polygonal-shaped odontogenic epithelial cells with nuclear hyperchromatism and pleomorphism are also identified (Figures 4D,G) and mentioned in three out of six NCLC-CEOT reports [16–18], but not in AR-COF reports. The amyloid-like products are distributed either associated with the epithelial nests or individually in the fibrous connective tissue stroma. No concentric laminated calcifications which form structures known as Liesegang rings are seen in both the NCLC-CEOT and AR-COF cases. Due to cortical bone perforation and gingival involvement are frequently noted in NCLC-CEOT/AR-COF cases, various degrees of chronic inflammatory cell infiltrates are frequently seen in the tumors (Figure 4E).

#### Immunohistochemical Findings

As NCLC-CEOT nomenclature indicated, numerous Langerhans cells immunostained by different markers, such as S-100 [9, 16, 18, 19], lysozyme [9], MT1 [9], LN3 [9], LKT6 [9], Langerin [18], and CD1a [17–20] and those detected by electron microscopy [9, 16], have been discovered in the epithelial nests in the NCLC-CEOT cases. Numerous Langerhans cells were also reported in the AR-COF cases [13–15, 21] with an approximately 40% mean ratio of positive Langerhans cells to tumor epithelial cells. The odontogenic epithelial cells in the NCLC-CEOT cases are positive for pan-cytokeratin [9, 16, 20], but are negative for epithelial membrane antigen (EMA) [16], involucrin [16], vimentin [16], collagen IV [16], and laminin [16]. The odontogenic epithelial cells in AR-COF cases and in two previously reported as NCLC-CEOT cases [15] have been shown to be positive for CK (AE1/AE3) [21], CK5/6 [21], CK19 [21], and CK10/13 [15], but are negative for CK7 [15] and CK8/18 [15]. In NCLC-CEOT cases, the amyloid-like products might or might not display green birefringence by polarized microscopy after Congo red staining [9, 16, 19, 20, 33]. By electron microscopy, these amyloid-like products consisted of dense accumulations of randomly oriented fibrillary materials, about 100 Å in thickness [16]. The amyloid-like products in the NCLC-CEOT cases were similar to the products in classic CEOT by electron microscopy. However, none of the NCLC-CEOT cases have used immunohistochemistry to prove that the amyloid-like products were ODAM (odontogenic ameloblast-associated protein). In AR-COF, the amyloid-like products were stained similarly as NCLC-CEOT cases [13, 15, 21]. The amyloid-like products have been further investigated and shown to be positive for ODAM protein immunohistochemically [13], which is the same protein found in tooth germs and in classic CEOT cases [34, 35].

### The NCLC-CEOT/AR-COF Cases Have Some Distinct Clinical and Radiographic Features as Compared With Classic CEOT and COF

The classic CEOT cases occur equally in male and female patients. The age range for CEOT patients is 4–78 years with an average age of 37.4 ± 18.3 years. The classic COF cases show a female predilection with a male to female ratio of approximately 1:2. The age range for COF cases is 8–63 years with a mean age of 32.7 ± 15.4 years. No significant difference was identified in the mean age or age range between the NCLC-CEOT, AR-COF, or NCLC-CEOT/AR-COF, and classic CEOT cases. However, there was a significant difference in the mean age between the NCLC-CEOT/AR-COF and classic COF cases (*p* < 0.01). The CEOT cases occur more frequently in the mandible than in the maxilla with a mandible to maxilla ratio of 1.5:1. The molar region of the mandible was commonly involved. The COF cases occurred slightly more frequently in the mandible than in the maxilla with a mandible to maxilla ratio of 1.3:1. The COF cases were also slightly more commonly discovered in the molar region of the jaw bone. There were significant differences in the tumor location and tooth involvement between the NCLC-CEOT/AR-COF and classic CEOT or COF cases (*p* < 0.001).

Except for the NCLC-CEOT cases, no palatal depression was mentioned in the classic CEOT cases. The swelling was the most common clinical chief complaint by the patients with CEOT. Both cortical bone disruption and cortical bone expansion were noted in about one-third of the CEOT cases. Although both the tooth displacement and root resorption were reported in the CEOT cases, the tooth displacement was more frequently seen than the root resorption. Except for AR-COF cases, palatal depression was identified in about one-third of the classic COF cases. The swelling was sometimes complained about by the patients with COF. The cortical bone expansion was more commonly noted than the cortical bone disruption in the COF cases. In the COF cases, the tooth displacement was also more frequently seen than the root resorption. No significant differences in the palatal depression, swelling, cortical bone disruption, or cortical bone expansion were identified between the NCLC-CEOT, AR-COF, or NCLC-CEOT/AR-COF, and classic CEOT or classic COF cases. However, there was a significant difference in the root resorption between the NCLC-CEOT/AR-COF and classic CEOT or COF cases (*p* < 0.001). Furthermore, a significant difference in the tooth displacement was noted between the NCLC-CEOT/AR-COF and classic CEOT cases (*p* < 0.01).

The overall recurrence rate of CEOT was about 11.6%. The recurrence rate in the COF cases was approximately 6.7%. No significant differences in the recurrence rate were found between the NCLC-CEOT, AR-COF, or NCLC-CEOT/AR-COF, and classic CEOT or classic COF cases.

### Overlapping Histopathological Features of Classic CEOT, NCLC-CEOT/AR-COF, and Classic COF Cases

Histopathologically, the classic CEOT cases have characteristic sheets or nests of polygonal odontogenic epithelial cells with marked intercellular bridges and occasional nuclear hyperchromatism and pleomorphism. In addition to epithelial nests, some amyloid-like materials and foci of calcifications dispersed in the fibrous connective tissue stroma are also present (Figure 4A). However, at the periphery of the CEOT cases, small inactive odontogenic epithelial nests admixed with some amyloid products in the fibrous connective tissue stroma, which mimicking the COF areas, can be identified (Figure 4B). Classic COF usually has small inactive odontogenic epithelial nests or strands dispersed in the myxofibrous connective tissue stroma. In NCLC-CEOT/AR-COF cases, in addition to the classic COF features, some or sometimes abundant amyloid-like materials are scattered or clustered adjacent to the epithelial islands or individually deposit in the fibrous connective tissue stroma (Figures 4C–H). Some nests of proliferating odontogenic epithelial cells with intercellular bridges and mild nuclear hyperchromatism and pleomorphism can sometimes be identified in the NCLC-CEOT cases (Figures 4D,E,G). Therefore, there were some overlapping histopathological features between the classic CEOT or NCLC-CEOT/AR-COF and classic COF cases.

## Discussion

### Calcifying Epithelial Odontogenic Tumor

Since CEOT is known as Pindborg tumor, we first reviewed the clinical and radiographic features of classic CEOT based on the review article by Pindborg [3]. The age range of overall CEOT cases is from 8 to 92 years with a mean age of 40 years. No obvious gender predilection is noted. Notably, most patients are Caucasian (73%). The mandibular molar region is the most common site. A mandible to maxilla ratio of 2:1 for the intraosseous cases and a predilection for anterior regions of both the mandible and the maxilla for the extraosseous cases have been reported. Radiographically, the smaller intraosseous CEOT lesions usually present as a unilocular radiolucency and the larger intraosseous CEOT lesions tend to show a multilocular radiolucency. Various amounts of radiopaque materials within the radiolucent area are characteristic features for CEOT. Approximately one-half of the intraosseous CEOT cases are associated with an unerupted tooth. A wide range of treatment methods, such as curettage, enucleation, excision, resection, mandibulectomy or maxillectomy, and radiation, have been reported for the treatment of CEOT cases. The recurrence rate is about 14%. Thus, a marginal resection is recommended as the treatment of choice for the CEOT cases. Recently, a large-scale review of 339 CEOT cases [32] has demonstrated similar epidemiologic findings as before. Half of the central CEOT cases show cortical bone perforation. The recurrence rate is approximately 11.6% for the central CEOT lesions. It is worth noting that seven cases of central LC variant CEOT cases were collected in this review article and no recurrence in the variant cases was noted in this review [32].

The CEOT has various histologic features [5]. Four histologic patterns have been described [6]. The most common and classic pattern is composed of sheets or nests of polyhedral epithelial cells with distinct intercellular bridges in the fibrous connective tissue stroma. Marked pleomorphism of the tumor epithelial cells is a typical feature but is not constantly found. The second pattern is composed of multiple spaces inside the epithelial sheets, giving a cribriform pattern. The third pattern shows scattered or densely populated epithelial cells with vacuoles in the cytoplasm, some giant tumor cells, and some mucoid substances in the stroma. The fourth pattern shows smaller nests and cords of epithelial cells with clear cytoplasm and dispersed in a dense fibrous stroma, resembling a salivary gland tumor. Although it is not clearly mentioned, the fourth histologic pattern is most likely present in the clear cell or Langerhans cell rich variants [7, 8]. Variable amounts and mixed histologic patterns within one tumor can be seen. Paucity or lack of calcifications [36–38] and the tumor composed of mainly small epithelial nests or islands [5, 6] have been reported in the CEOT cases. The NCLC-CEOT was first described by Asano in 1990 [9]. This histologic variant was later reviewed and included in Dr. Pindborg's review article with respect to the histopathological aspects of CEOT [5]. Later on, there is more LC variants of the CEOT cases have been reported [16–20, 39]. Interestingly, the majority of these NCLC variants of the CEOT cases were reported by scholars in the Asian countries [9, 16–19], except for one case [20]. So this unique LC variant of CEOT seems to have a predilection for the Asian people.

One of the characteristic histomorphological features of CEOT is amyloid or amyloid-like products. The amyloid or amyloid-like products are thought to be produced by the epithelial tumor cells. The amyloid-like substances have a smaller fiber size than conventional amyloid. An epithelial secretory product, a possible aberrant enamel matrix, is suspected. Further analysis of this amyloid-like protein in CEOT has been performed and this amyloid-like protein has been termed as ODAM [34, 40, 41], which is thought to be produced by developing tooth and the odontogenic epithelial cells in the CEOT. The origin of the tumor epithelial cells has been proposed as either cell from stratum intermedium of the tooth germs, reduced enamel epithelium of the closely related embedded tooth, or oral epithelium.

Dissection of the genetic alterations might be beneficial to establish the identity of certain lesions. Few genetic studies [42–44] have been performed on the classic CEOT cases. *Ameloblastin (AMBN)* gene mutation, a transversion (338A>T), and a transition (339G>A) through DNA sequencing of *AMBN* gene were identified in one CEOT sample in one study [43]. Another study sequencing the *PTCH1* gene revealed various single-nucleotide polymorphisms (SNPs) in the five CEOT cases [44]. Interestingly, the knockout mice for the *amelogenin (AMGN)* gene showed proliferative epithelium with dispersed concentric calcifications, which slightly mimics the histological features of human CEOT [45]. One recent study used the Ion AmpliSeq Cancer Hotspot Panel v2 which covered 2,856 mutations from 50 oncogenes and tumor suppressor genes and found a variety of amino acid changes among the different genes in five CEOT cases. These genes included *KDR, PTEN, TP53, KIT, MET, JAK3, PIK3CA, APC, and CDKN2A*. The majority of these gene mutations occurred in only one case each, thus these genes might not be the driver mutations for the CEOT. More samples are required to elucidate the genetic changes and define the roles of these genetic changes in the pathogenesis of the CEOT.

### Central Odontogenic Fibroma

Based on a recent large-scale review of COF [14], the COF has a female predilection with a female to male ratio of about 2:1. The age range is broad and equally distributed throughout the second to sixth decades, from 8 to 63 years, and the mean age is 33.9 years. The COF shows a nearly equal distribution in the maxilla and in the mandible. Interestingly, of maxillary COFs, 73% occur anterior to the first molar and 18% in the posterior maxilla. The mandibular lesions are mainly found in the posterior region (59%). The clinical presentation is usually an asymptomatic swelling between the tooth roots with a cortical bone expansion. Notably, approximately one-third of the maxillary COFs show palatal depression, which is an overlapping clinical feature like that in the NCLC-COET cases [18, 39]. In the radiographic findings, the majority of the COF cases show a well-defined radiolucent lesion and the ratio of the unilocular to multilocular COF lesions is about 2:1. The tooth displacement is seen in 55% of COF cases and the root resorption is discovered in 46% of COF cases.

In histological findings, the epithelial rich (WHO) type is the major type of COF which shows abundant odontogenic epithelial islands or cords dispersed in a collagenous fibrous connective tissue stroma. The fibrous connective tissue stroma is predominantly composed of fibromyxoid connective tissue. Dense collagen fibers, granular stroma, amyloid-like products, giant cells, and ossifications intermixed within the stroma are sometimes seen.

For the treatment of COFs, the majority of these cases are treated with conservative surgical excision with additional extraction of teeth, but one patient is reported to receive partial block resection. One out of 18 COF cases with follow-up data shows recurrence 3 years after initial surgical enucleation [14].

The origin of the COF is thought to be from the mesenchymal portion of the tooth germ or the periodontal membrane, thus COF frequently occurred in between the tooth roots [14]. No genetic alterations have been studied or reported for COF cases.

As our focus is on the AR-COF, which demonstrates overlapping features with NCLC-COET, the separated clinicopathological features are summarized below. Gardner was the first one who described the possible presence of amorphous eosinophilic materials within the COF [46]. Then, the amyloid rich variant of COF was first reviewed by Eversole in 2011 [13]. In this review, Langerhans cells highlighted by the CD1a immunostains were found in these AR-COF cases. The amyloid products in this variant were immunoreactive for ODAM protein, which is the same as in the CEOT cases. Due to lack of sheets of contiguous epithelial cells enmeshed in or surrounding more diffuse amyloid deposits in their cases, thus, AR-COF was favored over NCLC-CEOT in their diagnosis. In 2018, Zhou and Li [15] described the COF cases and particularly discussed their six AR-COF cases which had been previously reported as NCLC-COET cases by themselves in 2014 [18]. Based on different cytokeratin expression patterns and differences in amounts of Ki-67 and CD1a positive cells in the classic CEOT, NCLC-CEOT/AR-COF, and COF cases, they preferred re-categorizing their NCLC-CEOT cases as AR-COF cases. In a recent large-scale COF case series [14], 10 amyloid rich COF cases were included. Based on these studies, particularly focusing on the comparison results between the classic COF and AR-COF [13–15], it seems that the AR-COF showed overlapping features as classic COF; however, some distinct features are still noted in this review. The AR-COF cases were indeed have overlapped clinicopathological features with the NCLC-COET cases.

### Is NCLC-CEOT/AR-COF a Unique Disease Entity or in a Spectrum of COF/CEOT?

**Figure 5:**
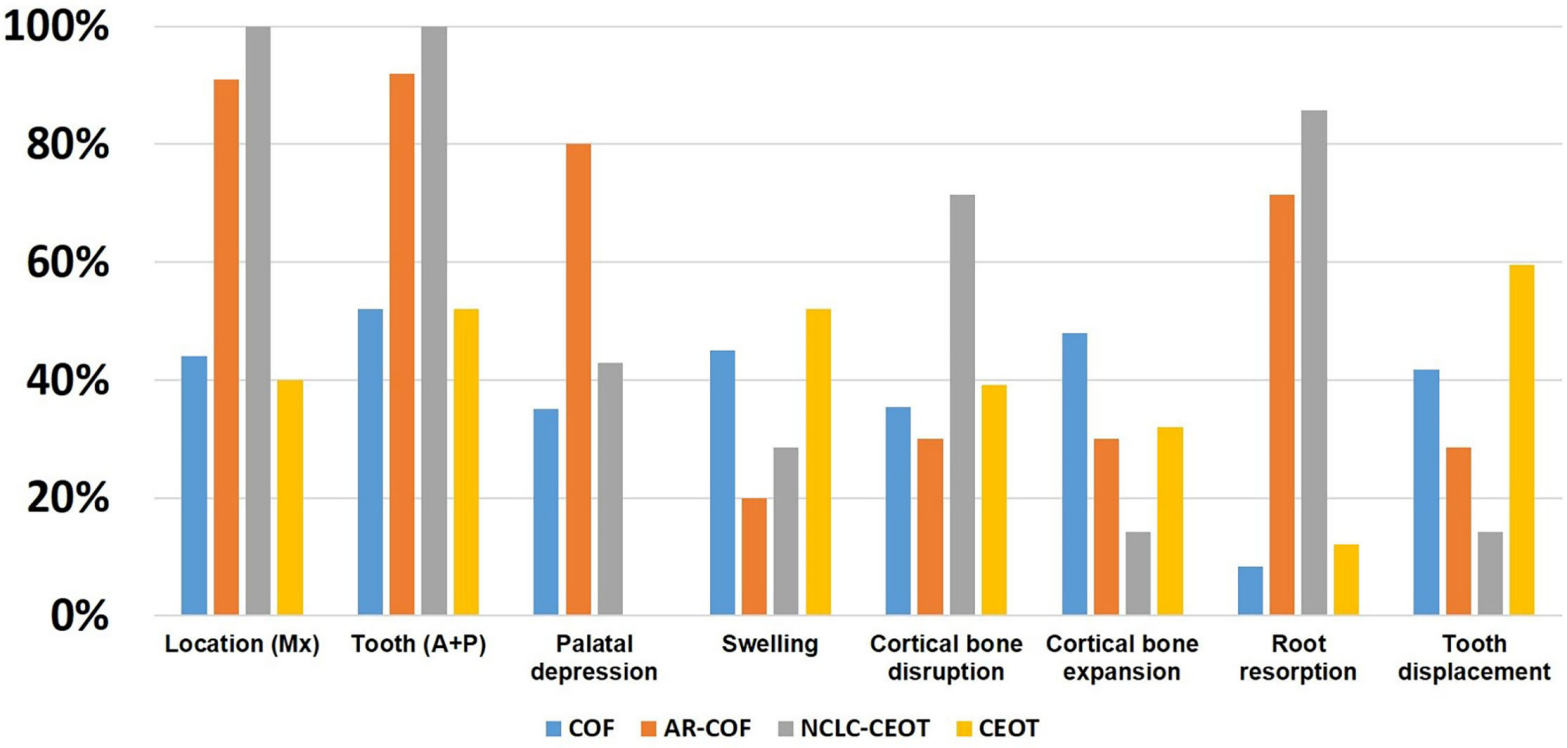
Comparison of the distribution of clinical findings in the COF, AR-COF, NCLC-CEOT, and CEOT cases.

The comparison of the clinical features of COF, AR-COF, NCLC-CEOT, and CEOT is summarized in Figure 5. As COF cases show frequent palatal depression, anterior maxilla involvement, inter-radicular location, very low recurrence rate, and small odontogenic epithelial nests microscopically, the NCLC-CEOT/AR-COF cases were thought to be a variant of COF [13, 15, 23]. Indeed, no palatal depression was particularly mentioned in the classic CEOT cases. Classic CEOT commonly occurred in the posterior mandible and was associated with an impacted tooth in one-half of the cases. These features were different from the NCLC-CEOT/AR-COF cases. However, as few COF cases have been reported, small amounts of amyloid products might be overlooked and the AR-COF cases account for 16% of the COF cases in the recent COF review [14], the clinical features of classic COF might be strongly affected by the AR-COF cases. Thus, we particularly reviewed the articles discussing the subtypes of COF or CEOT and including the detailed clinical information for further analysis. Interestingly, after excluding the AR-COF cases, more than one-half of the non-AR COF cases occurred in the mandible with nearly equally involved the anterior and posterior mandible (Figure 5). Although the posterior mandible is thought to be the most common site for the classic CEOT, non-NCLC CEOT cases showed maxilla: mandible ratio of 2:3 and nearly equal anterior and posterior teeth involvement. Swelling and cortical bone expansion than the palatal depression and cortical bone disruption were more frequently seen in the non-AR COF and non-NCLC CEOT cases. It is worth noting that both the CEOT and COF cases tend to cause tooth displacement rather than root resorption, but approximately three-fourths of the NCLC-CEOT/AR-COF cases show root resorption, which may be a misleading factor resulting in the diagnosis of NCLC-CEOT/AR-COF as an aggressive disease clinically. Although the NCLC-CEOT/AR-COF cases share some similar features as “overall” COF cases, after subtracting the AR-COF cases from the overall COF cases, the NCLC-CEOT/AR-COF seems to be a more distinct disease entity from the non-AR COF cases.

**Figure 6:**
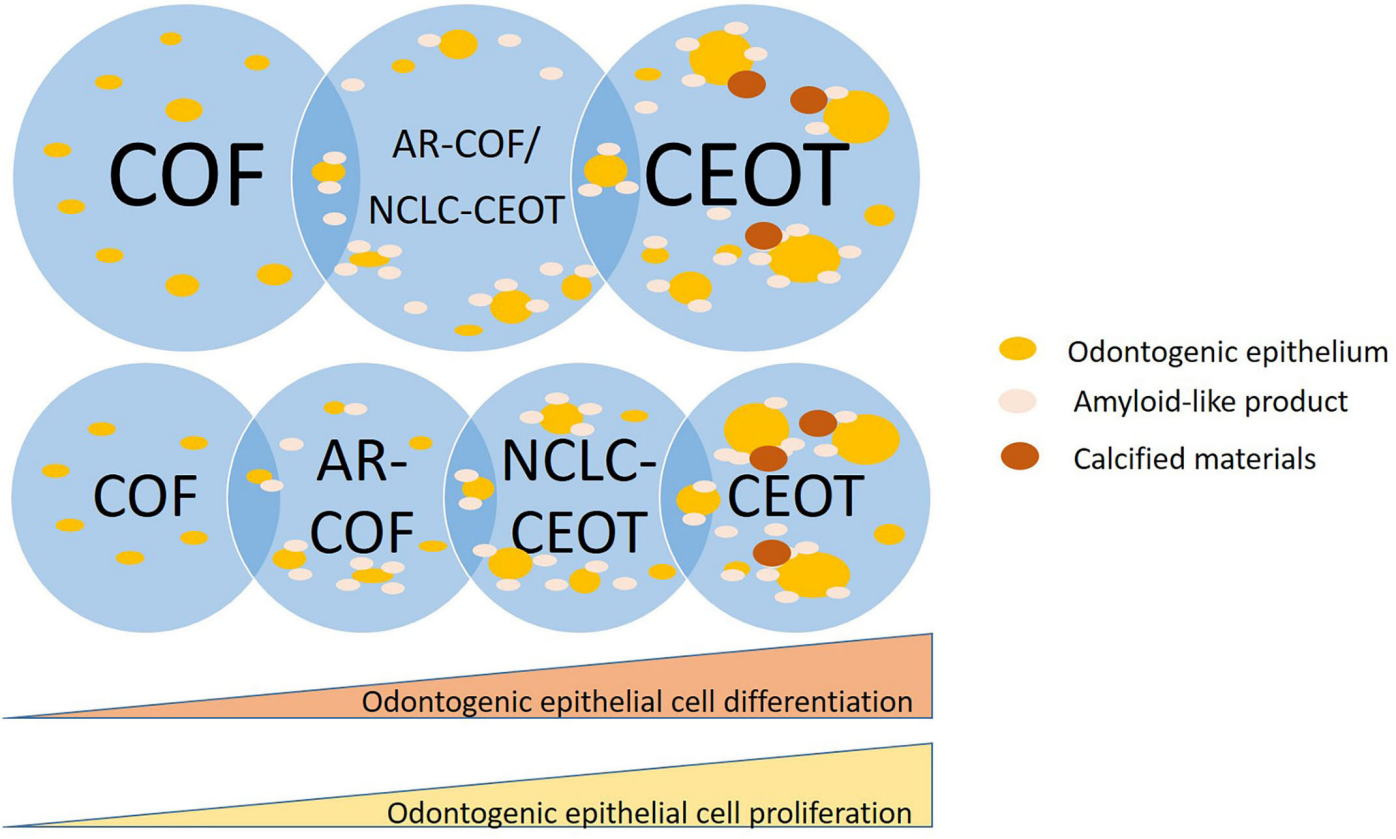
Schematic diagram of NCLC-CEOT/AR-COF is a unique disease entity or in a spectrum of COF/CEOT.

Whether NCLC-CEOT/AR-COF should be categorized as AR-COF, separated as a unique disease entity or it is a spectrum of COF/CEOT is still in debate [23, 24]. In detailed histopathological examinations of classic and variants of CEOT and COF, we have taken photographs from different areas of the tumors which are shown in Figure 4. Based on this series of histologic photographs, the overlapping histopathological features at the periphery of CEOT and COF can be observed. The proliferation of odontogenic epithelial cells rather than the usual inactive status can be seen in the NCLC-CEOT cases. Based on the recent study [47], such abundant proliferating epithelial components are away from the inactive status and can hardly be ignored in the diagnosis of these NCLC-CEOT/AR-COF cases. The diagnosis of COF, a mesenchymal tumor, seems not to be representative of the histopathological features of the disease possessing the proliferating odontogenic epithelial cells. This controversy in diagnosis has also been reported [24] and encountered even after Dr. Pindborg had defined CEOT and still included one NCLC-CEOT case in his review article describing various CEOT histopathologic patterns [5]. It is also possible that NCLC-CEOT/AR-COF is in the COF/CEOT spectrum and has a unique clinical presentation due to its specific tumor location (Figure 6). From our point of view, an abundance of odontogenic epithelium in AR-COF contradicts its current classification as a tumor of pure mesenchymal origin, let alone the presence of amyloid-producing odontogenic epithelial cells in this entity in the discussion. The capability of protein secretion advocates that the active metabolic status of the epithelial cells rather than the inactive state is accepted currently in the COF cases. Further, the molecular biological studies to characterize these diseases may shed light on the answer to this question.

## Conclusion

The NCLC-CEOT and AR-COF cases share similar clinicopathological features and they are most likely to be the same disease entity. Due to nearly no recurrence for the NCLC-CEOT/AR-COF cases, conservative surgical treatment is recommended. The NCLC-CEOT/AR-COF cases show some overlapping clinicopathological features with COF rather than CEOT. However, the differences in some clinicopathological features are also recognized between the NCLC-CEOT/AR-COF or COF and CEOT cases. It is still under debate whether the NCLC-CEOT/AR-COF is a unique disease entity or belongs to either CEOT or COF or even in a spectrum of COF/CEOT.

## Author Contributions

JC, Y-PW, C-PC, and Y-SC: conceptualization and writing, review, and editing. C-HT, P-HL, and JC: methodology and investigation. JC: validation, funding acquisition and resources, and supervision. C-HT and JC: formal analysis, writing, and original draft preparation. C-HT, P-HL, Y-PW, and Y-SC: data curation. Y-SC and JC: visualization. All authors have read and agreed to the published version of the manuscript.

## Funding

This research was funded by Grant Nos. 109-S4770, 108-N4355, 107-N4024, and 106-N3729 from the National Taiwan University Hospital, Grant Nos. MOST 109-2314-B-002-040, MOST 108-2314-B-002-039, and MOST 106-2314-B-002-053 from the Ministry of Science and Technology, Executive Yuan, Taiwan, ROC, and Grant No. NHRI-EX109-10612EC from the National Health Research Institutes, Taiwan, ROC.

## Conflict of Interest

The authors declare that the research was conducted in the absence of any commercial or financial relationships that could be construed as a potential conflict of interest.

## Publisher's Note

All claims expressed in this article are solely those of the authors and do not necessarily represent those of their affiliated organizations, or those of the publisher, the editors and the reviewers. Any product that may be evaluated in this article, or claim that may be made by its manufacturer, is not guaranteed or endorsed by the publisher.
